# A New Understanding of the Mechanism of Injury to the Pelvis and Lower Limbs in Blast

**DOI:** 10.3389/fbioe.2020.00960

**Published:** 2020-08-13

**Authors:** Iain A. Rankin, Thuy-Tien Nguyen, Diagarajen Carpanen, Jonathan C. Clasper, Spyros D. Masouros

**Affiliations:** ^1^Department of Bioengineering, Imperial College London, London, United Kingdom; ^2^Department of Trauma and Orthopaedic Surgery, Frimley Park Hospital, Frimley, United Kingdom

**Keywords:** biomechanics, traumatic amputation, fracture, blast injury, military, mouse, soil, sand

## Abstract

Dismounted complex blast injury (DCBI) has been one of the most severe forms of trauma sustained in recent conflicts. This injury has been partially attributed to limb flail; however, the full causative mechanism has not yet been fully determined. Soil ejecta has been hypothesized as a significant contributor to the injury but remains untested. In this study, a small-animal model of gas-gun mediated high velocity sand blast was used to investigate this mechanism. The results demonstrated a correlation between increasing sand blast velocity and injury patterns of worsening severity across the trauma range. This study is the first to replicate high velocity sand blast and the first model to reproduce the pattern of injury seen in DCBI. These findings are consistent with clinical and battlefield data. They represent a significant change in the understanding of blast injury, producing a new mechanistic theory of traumatic amputation. This mechanism of traumatic amputation is shown to be high velocity sand blast causing the initial tissue disruption, with the following blast wind and resultant limb flail completing the amputation. These findings implicate high velocity sand blast, in addition to limb flail, as a critical mechanism of injury in the dismounted blast casualty.

## Introduction

Blast injury was the leading mechanism of wounding and death in recent military conflicts, and its incidence in the civilian setting has also increased steadily over the last 40 years (Mcfate and Moreno, 2005; Edwards et al., 2016). Improvised explosive devices have risen as the weapon of choice for inflicting blast injury, consisting of roadside explosives and mines, explosive formed projectiles, and suicide bombings (Ramasamy et al., 2009). These principally result in extremity wounding, for which the burden of injury can be substantial (Griffiths and Clasper, 2006; Owens et al., 2007). Dismounted complex blast injury (DCBI) is one of the most severe patterns of injury in the dismounted (on-foot) casualty. It consists of traumatic amputation of at least one lower limb, a severe injury to another limb, pelvic, perineal and/or abdominal trauma, with extensive soft tissue damage (Ficke et al., 2012). Of the DCBI injuries described, battlefield data have shown pelvic vascular injury to be the single greatest predictor of mortality (Rankin et al., 2020). Post-mortem CT data have shown unstable pelvic fractures with lateral displacement of the sacroiliac joints to be the greatest predictor of vascular injury (Rankin et al., 2020). When these injuries are observed in combination with traumatic amputation and significant perineal injury, then the highest risk of mortality is seen (Rankin et al., 2020).

Displaced pelvic fractures with vascular injury secondary to a shock-tube mediated blast wave have been reproduced in an animal model, which linked this pattern of injury to outward flail of the lower limbs (Rankin et al., 2019). The authors, however, noted a lack of traumatic amputation and perineal injury with this model and suggested a further mechanism of injury was required to produce the pattern of injury seen in DCBI. The mechanisms of injury following any explosion can be divided into four categories: primary (effects of the blast wave over-pressurization), secondary (penetrating injury due to energized projectiles), tertiary (displacement of the body due to the blast wind), and quaternary (miscellaneous including burns) (Webster and Clasper, 2016). Whilst tertiary is implicated in pelvic injury through limb flail, the mechanism of injury resulting in traumatic amputation and perineal injury is not clearly understood. Traumatic amputation in the dismounted casualty has been hypothesized to occur due to a combination of primary and tertiary blast mechanisms; fracture of the long bone from the blast wave followed by the blast wind completing the amputation (Hull and Cooper, 1996). Other authors have indicated tertiary blast alone to be implicated, as lower limb flail propagated by the blast wind results in amputation. No consensus has been reached on the mechanism of injury for traumatic amputation and no model has reproduced the perineal or abdominal injuries seen in blast.

Across all blast injury mechanisms, propelled energized fragments are the most common wounding modality seen in recent conflicts (Covey and Ficke, 2016; Edwards and Clasper, 2016). These energized fragments may be from the explosive device itself or objects from the surrounding environment. Whilst secondary blast injury from energized fragments has been clearly identified as a significant contributor to mortality, the contribution of energized environmental debris (soil, sand and gravel) to injury patterns of the DCBI casualty is not known (Covey and Ficke, 2016).

The method by which soil propagates following a land mine or buried IED blast is known. Upon detonation, buried explosive devices generate a shockwave which compresses the surrounding soil. Gas from the explosion is released at high velocity and acts to eject this soil, propelling it at supersonic speeds of up to 900 m/s (depending upon soil characteristics and explosive mass) (Tremblay et al., 1998). The energized fragments subsequently rapidly decelerate to 600 m/s or less before impacting casualties (Bowyer, 1996). The direction of expansion of the soil ejecta is heavily dependent on the soil’s properties; the result, however is typically an inverse cone with a projection angle of between 45 and 120 degrees (Grujicic et al., 2008). Upon impact, the physical momentum transfer from the soil ejecta is likely to cause displacement and produce significant injury to the dismounted casualty. The process by which the casualty gets injured has not been investigated in a physical model.

Accordingly, the aims of this study were (1) to replicate impact from propelled high velocity soil as occurs following blast in a small animal mouse model, utilizing a gas-gun system, and (2) to investigate the effect of increasing velocity on the resulting injury pattern. Our hypothesis was that high velocity soil ejecta would contribute to the injury pattern seen in DCBI and play an essential role in both soft tissue and skeletal injury.

## Materials and Methods

The experimental design and procedures were carried out in compliance with the UK Animal (Scientific Procedures) Act 1986. Testing was conducted using an established model on fresh-frozen cadaveric male MF-1 (out-bred, ex-breeder, wild type) murine specimens (8–9 weeks of age, median weight 35.5 g (range 31.2–40.5 g, *n* = 22), Charles River Ltd., United Kingdom) (Rankin et al., 2019). Specimens were stored at −20°C and thawed at room temperature (21 ± 2°C) for 2–4 h prior to testing.

**FIGURE 1:**
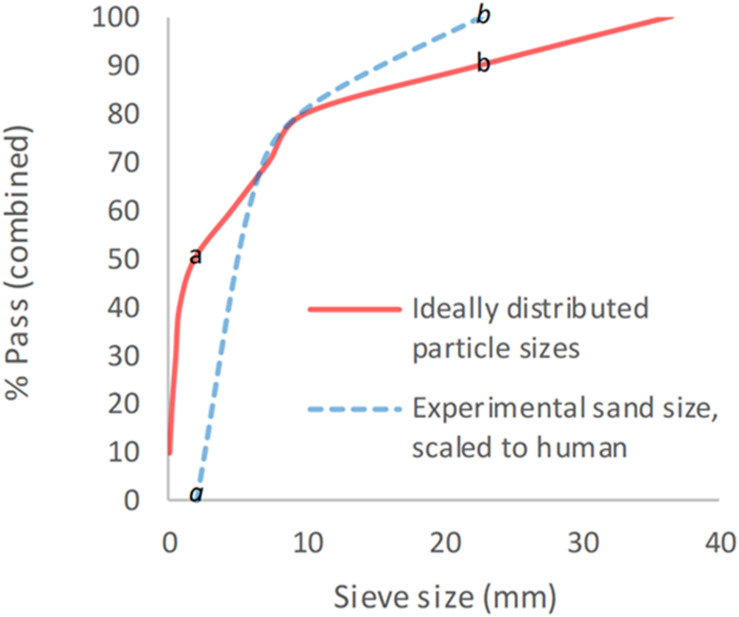
Experimental sand sizes used, scaled to human values, shown alongside ideally distributed particle sizes. a = human median value. b = human 85th centile. *a* = lower limit of experimental sand range. *b* = upper limit of experimental sand range. % pass (combined) describes the percentage of total volume of sand passing a specific sieve size; sieve size (mm) relates to the diameter of each hole within the sieve.

Sand size and properties were chosen based upon NATO unclassified AEP-55 recommendations for typical sandy gravel soil granulometry (NATO/PFP Unclassified, 2006). The sand size distribution was subsequently scaled to the murine model based upon recommended animal scaling parameters in blast, where the scale is equal to the length of a parameter of the human species divided by that of the animal species used (λ_*L*_ = L_1_/L_2_) (Panzer et al., 2014). The thigh circumference of each species was taken as the representative parameter for scaling, in view of traumatic amputation of the lower limb being a primary outcome. Median mouse thigh circumference was calculated as 2.7 cm (range 2.6–3.1 cm) from specimens (*n* = 22), whilst human thigh circumference was taken from literature as 55 cm (White and Churchill, 1971). From this, an upscaling of 20× for sand size was utilized (λ_*L*_ = 55/2.7 = 20). A minimum sand size cut-off of 0.1 mm was taken to avoid sublimation of sand particles smaller than this at high velocity. A sandy gravel aggregate size range as closely representative to human scaled values was subsequently chosen, ranging from the human ideal particle size median value to the 85th centile value, consisting of 60% sandy gravel sized 0.1–0.3 mm, 20% sized 0.3–0.5 mm, and 20% sized 0.5–1 mm. The experimental sand sizes and distribution used (scaled to human values) are shown alongside those recommended in NATO AEP-55, ideally distributed particle sizes in Figure 1 (NATO/PFP Unclassified, 2006).

The sand was housed within a hollow polycarbonate sabot which was loaded into the firing chamber of a double-reservoir gas-gun system (Nguyen et al., 2018). Within this system, a 2-l reservoir charged with air or helium and a Mylar^®^ diaphragm firing mechanism was used to accelerate the sabot-sand unit down a 3-m-long, 32-mm-bore barrel. The output velocity, which can range between 20 and 600 m/s, was controlled by the thickness of the Mylar^®^ diaphragm. To accelerate the sabot-sand unit to the desired velocity, the reservoir section of the gas gun was charged to a predetermined firing pressure. The pressure was maintained within the reservoir section by a Mylar^®^ diaphragm of appropriate thickness (ranging from 50 to 150 μm). The system utilizes a priming section, which is charged to a pressure below the rupture pressure of the diaphragm. This reduces the pressure gradient across the mylar diaphragm (containing the reservoir system) and prevents it from rupturing early, as the reservoir is filled. At the point of initiating firing of the gas gun, the pressure in the prime section is vented, resulting in rupture of the diaphragm, with release of the pressurized gas. This accelerates the sabot-sand unit down the barrel to exit into the target chamber, where the sabot is separated from the sand by a sabot-stripper constructed from aluminum and polycarbonate slabs and a heavy stainless-steel block. The sabot is halted at this point, while the sand continues to travel toward the murine specimen at the intended terminal velocity.

**FIGURE 2:**
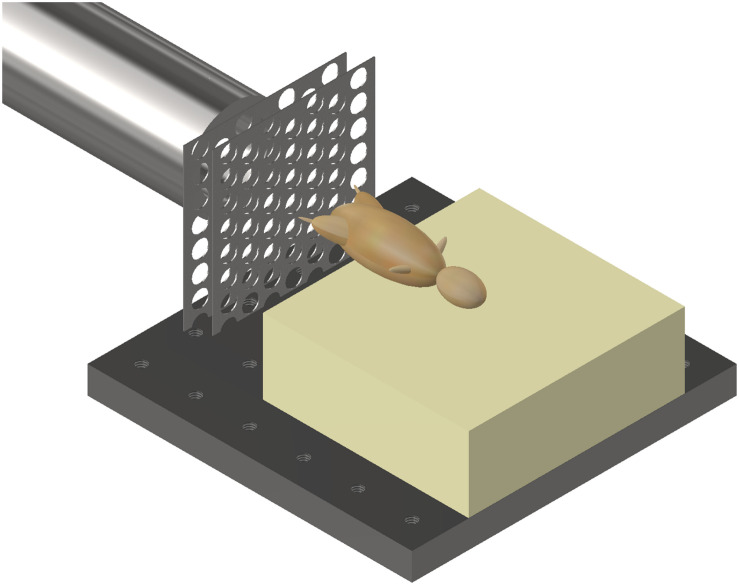
Gas gun with under-body sand blast mounting platform, fenestrated steel fences, and mouse. Mouse represented with model.

**FIGURE 3:**
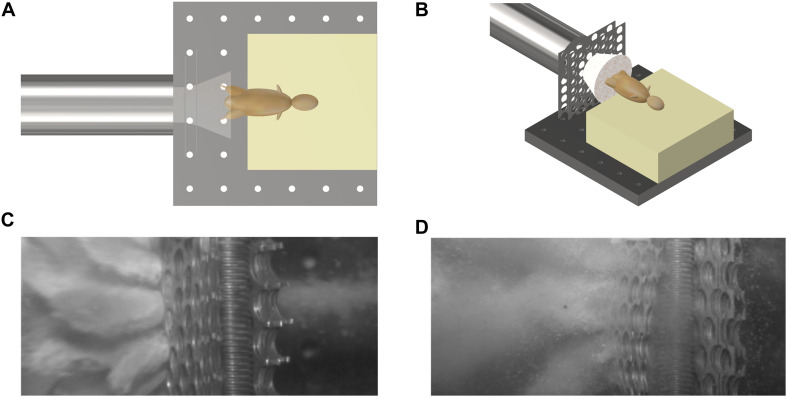
**(A)** Aerial view of schematic illustrating initial sand stream passing through offset fenestrated steel fences causing dispersion of the sand prior to impact with the specimen. **(B)** Oblique view of schematic illustrating initial sand stream passing through offset fenestrated steel fences causing dispersion of the sand prior to impact with the specimen. **(C)** Photograph showing the initial sand stream converted into multiple streams. **(D)** Photograph showing multiple streams dispersing into a widely distributed spread of high velocity sand.

Mice were secured in a supine posture on a polyurethane foam mount within the target chamber. A single cable tie across the thorax was applied to secure the specimens in position on the mount, whilst leaving the pelvis and lower limbs exposed. In order to simulate the sand ejecta spread, two interconnecting fenestrated steel fences, separated by 5 mm and offset to one another by 50% of the diameter of each fenestration, were placed distal to the gas-gun outlet and 50 mm proximal to the mount (Figure 2). Offsetting of the fenestrated steel fences changed the initial stream of sand delivered by the gas gun into multiple individual streams of differing trajectories, which subsequently dispersed into a widely distributed spread of high velocity sand. Figures 3A,B illustrate this setup in the aerial and oblique views respectively. Figure 3C shows a photograph of the initial sand stream being converted into multiple streams, followed by Figure 3D which shows the sand dispersing into a widely distributed spread of high velocity sand.

The speed of the sand particles at the point of impact with the sample was estimated using high-speed photography (Phantom VEO710L, AMETEK, United States) at 68,000 fps. An average velocity for the sand cloud as a whole was determined based upon identifying and tracking four unique points spread across the distributed sand. These points varied in velocity and were chosen from the front, front-center, center, and center-back of the peripheries of the sand spread. From this, the mean with standard deviation of the velocity of the sand spread as a whole was calculated.

Following these experiments, a single control test was performed utilizing the maximum gas-gun pressure used previously with the absence of any sand ejecta. This was performed in order to ascertain whether any injurious effects are caused by the pressurized air alone. This control test was performed on a single control mouse specimen.

Prior to and following each test, mouse specimens underwent radiographic imaging using a mini C-arm (Fluoroscan^®^ InSight^TM^ FD system, United States) to identify any fractures in the specimen and assist with injury classification. Subsequent to this, specimens underwent dissection to identify injury patterns. Recorded injury patterns included; (1) lower limb degloving; (2) soft tissue pelvic and perineal injury [the Faringer system was used to classify the location of the soft-tissue injury anatomically: zone I (perineum, anterior pubis, medial buttock, posterior sacrum), zone II (medial thigh, groin crease), or zone III (posterolateral buttock, iliac crest)] (Faringer et al., 1994); (3) lower limb traumatic amputation; (4) open abdominal injury; and (5) pelvic fracture. Pelvic fractures were classified in accordance with the Tile criteria (Tile, 1996). Where a lower limb open fracture was present with extensive soft tissue loss, the injury was classified as a traumatic amputation.

### Statistical Analysis and Development of the Risk Function

The NCSS statistical software was used for statistical analysis (version 12, Utah, United States). A likelihood-criteria best-fit analysis, with the aid of probability plots, was performed to choose the distribution that best fit the data for each injury type. The Weibull distribution was shown to be the best fit in the majority of cases; hence, it was chosen as the probability distribution to represent the risk for all injury types observed in this study. Weibull survival analysis was used to examine the association between sand velocity and each category of injury. The Weibull regression model is *P*(*v*) = 1−*e*^−(*v*/λ)^κ^^, where *P* is the probability of injury, *v* (the average velocity of sand) is the predictor variable, and λ and κ are the corresponding coefficients associated with the predictor variable. To derive the survivability curves, data were classified as left censored where injury was present and right censored where there was no injury. The normalized confidence interval size (NCIS) of the survivability curves was determined as the ratio of the width of the CI to the magnitude of the predictor variable at a specific risk level.

## Results

### Replication of Impact With High Velocity Soil Ejecta

**FIGURE 4:**
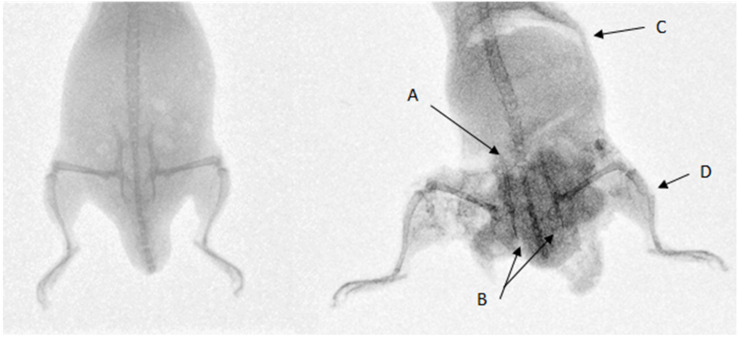
Left: uninjured mouse. Right: mouse injured with sand blast at 252 m/s sustaining pelvic fractures with **(A)** sacroiliac joint disruption and **(B)** pubic rami fractures, **(C)** abdominal injury with free air in the abdomen, perineal injury, and **(D)** an open tibial fracture with surrounding extensive soft tissue loss. The increased density on the injured mouse represents sand debris.

Twenty-two cadaveric mice were used, including one control specimen. No injuries were seen in the control specimen. Average sand velocity ranged from 166 ± 12 m/s to 271 ± 24 m/s. Radiographs showing an uninjured mouse next to a mouse injured by sand blast are shown in Figure 4. Table 1 details the types of injuries seen across all mice. Table 2 details the pelvic fracture patterns sustained. Supplementary Table 1 details the injuries sustained by each individual mouse, the associated velocity of sand, and the gas-gun pressures utilized to achieve the sand velocity. Supplementary Video 1 shows a high-speed video capturing sand impact at 208 m/s.

**TABLE 1:** Types of injuries sustained across all mice.

**Total number of mice^a^**	**Injured mice**	**Lower limb degloving injuries**	**Soft tissue pelvic and perineal injuries**	**Including Faringer zones^b^**			**Open abdominal injuries**	**Traumatic amputations**	**Pelvic fractures**
				**1**	**2**	**3**			
21	16 (76%)	14 (67%)	14 (67%)	11	12	5	8 (38%)	7 (33%)	5 (24%)

**^*a*^Excluding control specimen. ^*b*^The Faringer system was used to classify the location of the soft-tissue injury anatomically: zone I (perineum, anterior pubis, medial buttock, posterior sacrum), zone II (medial thigh, groin crease), or zone III (posterolateral buttock, iliac crest) (Faringer et al., 1994).**

**TABLE 2:** Pelvic fracture patterns sustained.

**Pelvic fractures**	**Tile classification^a^**	**Pubic rami**	**Pubic symphysis disruption**	**Acetabulum**	**Iliac wing**	**Sacrum**	**Sacroiliac joint disruption**
5	5 (100%) Type C	5 (100%)	0 (0%)	1 (20%)	3 (60%)	1 (20%)	4 (80%)

**^*a*^Pelvic fractures were classified in accordance with the Tile criteria: Type A (pelvic ring stable), Type B (pelvic ring rotationally unstable, vertically stable), and Type C (pelvic ring rotationally and vertically unstable).**

### Effects of Increasing Velocity on Injury Patterns

**FIGURE 5:**
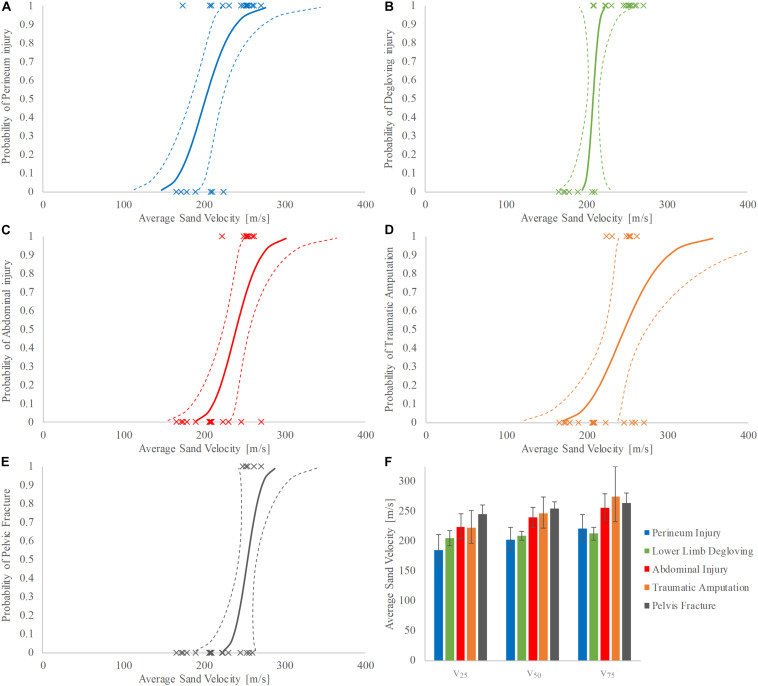
**(A–F)** Injury risk curves for perineum injury **(A)**, lower limb degloving **(B)**, abdominal injury **(C)**, traumatic amputation **(D)**, and pelvic fracture **(E)** as a function of average sand velocity; 95% CI is represented with dashed lines. **(F)** Shows the respective v_25_, v_50_, and v_75_ for each category of injury; 95% CI is represented with variability whiskers.

Increasing velocity produced injury patterns of worsening severity. The velocity at 50% risk of injury (v_50_) for soft tissue pelvic and perineal injury was 202 m/s (95% confidence interval (CI): 183–223 m/s, normalized confidence interval size (NCIS): 0.20) (Figure 5A), for lower limb degloving was 208 m/s (95% CI: 202–216 m/s, NCIS: 0.07) (Figure 5B), for open abdominal injury was 239 m/s (95% CI: 223–257 m/s, NCIS: 0.14) (Figure 5C), for traumatic amputation was 247 m/s (95% CI: 222–274 m/s, NCIS: 0.21) (Figure 5D), and for pelvic fracture was 254 m/s (95% CI: 243–265 m/s, NCIS: 0.09) (Figure 5E). The NCIS of all injury curves for v_50_ were found to be low, at less than 0.25. Full injury risk curves with 95% CIs are shown in Figures 5A–E, with the 25, 50, and 75% risks of injury presented as bar graphs in Figure 5F (v_25_, v_50_, and v_75_, respectively).

## Discussion

The first aim of this study was to replicate secondary blast injury caused by high velocity sand blast in a mouse model using a gas-gun system. We hypothesized that high velocity sand blast causes extensive soft tissue and skeletal disruption and plays an essential role in the injury pattern seen in DCBI. The pattern of injury in DCBI involving traumatic amputation of at least one lower limb, a severe injury to another limb, pelvic, urogenital, and/or abdominal trauma was reproduced in our model as predicted (Figure 4; Ficke et al., 2012).

Additionally, progressively worsening severity of injuries was seen with increasing sand velocity. Lower speeds were associated with soft tissue disruption to the perineum and lower extremities whilst higher speeds resulted in open abdominal injury, traumatic amputation, and pelvic fracture. The injury curves presented (Figures 5A–E) show a clear link between increasing sand velocity and likelihood of injury, with each curve demonstrating low NCIS at the 50% probability of injury.

All pelvic fractures sustained in this study were rotationally and vertically unstable, correlating with battlefield and clinical data. Pelvic fractures secondary to blast in the dismounted casualty are inherently unstable in nature, consisting of predominately pubic symphysis and sacroiliac joint disruption followed by pubic rami, sacral and acetabular fractures (Oh et al., 2016; Webster et al., 2018; Rankin et al., 2019). A previous mouse model demonstrated a link between shock-tube mediated outward flail of the lower limbs and displaced pelvic fractures with vascular injury (Rankin et al., 2019). These fractures consisted predominately of pubic symphysis and sacroiliac joint disruption, with minimal rami, sacral, or acetabular fractures (Rankin et al., 2019). The authors did acknowledge that a limitation of the study was the lack of secondary blast injury, which they hypothesized would worsen the injuries seen. In the current study, high velocity sand has recreated secondary blast injury in the mouse model, which has resulted in pelvic fractures predominately at the pubic rami, with posterior disruption at the sacroiliac joints or iliac wing, and sacral or acetabular fractures. Notably, no pubic symphysis disruption was seen. The combination of the findings in these two studies consequently allow us to explain fully the mechanism of pelvic injury of the dismounted casualty: lower limb flail (tertiary blast injury) results principally in pubic symphysis and sacroiliac joint disruption with vascular injury whilst high velocity sand blast (secondary blast injury) results principally in pubic rami fractures (with associated posterior pelvic disruption), sacral and acetabular fractures. This mechanism of injury explains the observation from battlefield data and suggests that pelvic fractures seen following dismounted blast are due to both secondary (sand blast) and tertiary (lower limb flail) blast-injury modalities (Oh et al., 2016; Webster et al., 2018; Rankin et al., 2019).

Lower limb flail (tertiary blast injury) has been hypothesized to cause pelvic bony displacement following the initial fracture with subsequent displacement of the intrapelvic soft tissues causing pelvic vascular injury (Rankin et al., 2019). Military clinical data have shown that pelvic vascular injury occurs predominately at the posterior pelvis, with significant retroperitoneal bleeding (Rankin et al., 2020). It was identified as the injury with the single greatest risk of mortality in the dismounted pelvic blast injury casualty, followed by traumatic amputation (Rankin et al., 2020). Whilst not explored further in this study, traumatic amputation presents with vascular injury both at the zone closest to the blast (where widespread damage and anatomical destruction is present) and at a zone more proximal to this, with lacerations of small and large blood vessels. These vascular injuries proximal to the zone of destruction result in surgical amputation being subsequently required at a level higher to the zone of traumatic amputation (Clasper and Ramasamy, 2013). Furthermore, surgical amputations may be required in cases where a tensile stretching injury to the major vasculature of the extremity has been applied during limb flail, or where a soft tissue injury (without traumatic amputation) following sand blast has resulted in vascular injury. As such, the injury risk threshold for traumatic (and subsequently required surgical) amputation may be under-represented in the present study.

**FIGURE 6:**
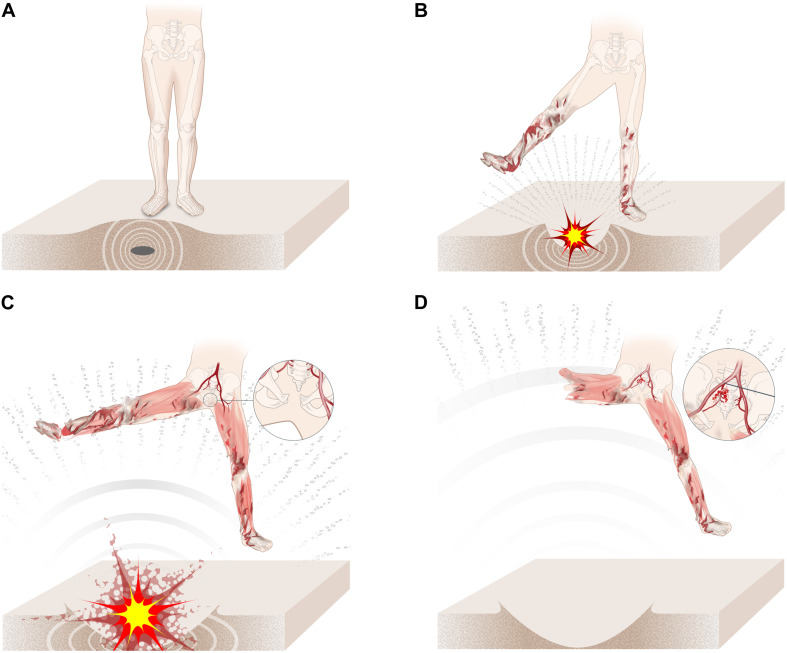
**(A–D)** The mechanism of injury of dismounted blast trauma. **(A)** Casualty stands on an IED which detonates, causing the initial blast wave to compress the surrounding soil. **(B)** Sand is ejected at high velocity toward the casualty, causing soft tissue degloving and skeletal disruption. **(C)** The casualty is impacted by the blast wind, resulting in lower limb flail with separation of the pubic symphysis. **(D)** The blast wind completes the amputation at the level of the initial disruption, whilst continued leg flail results in opening of the sacroiliac joint and vascular injury.

Several mechanisms of injury for blast-related traumatic amputation have been described. This was first hypothesized to be due to a combination of the initial blast wave (primary blast injury) resulting in diaphyseal fracture to the long bones of the femur or tibia, with the subsequent blast wind (tertiary blast injury) resulting in separation and amputation of the limb (Hull and Cooper, 1996). More recent data have contested this mechanism: review of post-mortem CT data from recent conflicts showed no link (as previously described) between traumatic amputation and primary blast lung injury and a higher rate of through-joint traumatic amputation than previously seen, which is an injury pattern not explained by the shock-wave mechanism of injury (Singleton et al., 2014). The authors suggested lower limb flail (tertiary blast injury) in isolation as an independent mechanism for blast-mediated traumatic amputation. Limb flail has been shown in an animal model to be linked to traumatic amputation (Rankin et al., 2019). However, the traumatic amputation rates seen in the animal study were far lower than what is seen in battlefield data (Rankin et al., 2019, 2020). When a pre-test crush was applied to the thigh causing soft tissue disruption, all mice subsequently sustained traumatic amputations following lower limb flail in simulated blast-wave conditions (Rankin et al., 2019). The authors hypothesized that the lower-than-expected traumatic amputation rates were due to the absence of secondary blast injury causing an initial disruption to the soft tissues of the thigh. In the current study, traumatic amputation was seen to occur at high velocities (v_50_ traumatic amputation: 247 m/s, 95% CI: 222–274 m/s), whilst soft tissue disruption alone (lower limb degloving) was present at lower velocities (208 m/s, 95% CI: 202–216 m/s). Previous research linked an initial injury to the soft tissues of the thigh to subsequent traumatic amputation following lower limb flail; based on this research, it may be inferred that the combination of sand blast with limb flail would likely result in traumatic amputation at lower velocities (Rankin et al., 2019). Whilst sand blast in isolation is sufficient to cause traumatic amputation, it is unlikely to be experienced in isolation in an explosion. As such, we propose the following novel mechanism of injury causing traumatic amputation in the dismounted casualty: an initial secondary blast injury (high velocity sand blast) causes disruption to the soft tissues of the limb, with or without skeletal disruption, following which the blast wind and resultant limb flail (tertiary blast injury) complete the traumatic amputation at the level of the disruption (Figures 6A–D). Whilst environmental debris following blast is linked to infection and delayed amputation, high velocity sand blast has not been implicated previously as a causative component of traumatic amputation in the dismounted casualty (Khatod et al., 2003; Covey and Ficke, 2016). These mechanisms of injury of dismounted blast trauma, resulting in pelvic fracture and traumatic amputation, are illustrated in Figures 6A–D.

The initial velocity of sand blast reaches up to 900 m/s following the initial energy from the explosion, but rapidly decelerates to 600 m/s or less before impacting casualties (Bowyer, 1996; Tremblay et al., 1998). In the current study, the v_50_ for sand blast to cause traumatic amputation in the mouse model was 247 m/s (95% CI: 222–274 m/s), with a sand size when scaled to the human ranging from 2.0 to 20 mm. No comparable human research has been performed previously with which to evaluate these findings. Research investigating the risk of fracture to human cadaveric tibiae when impacted by a gas-gun delivered 4.5 mm fragment simulating projectile, however, has shown that similar velocities resulted in fracture: the v_50_ for fracture was shown to be 271 m/s (95% CI: 241–301 m/s) (Nguyen et al., 2020). No previous research has quantified the risk of soft tissue injury (degloving, perineal or open abdominal injury) or pelvic fracture caused by energized soil or fragments.

Several factors must be taken into consideration when inferring the results and conclusions of these findings in the mouse model, for subsequent interpretation to human injury risk. Previous work has described in detail the suitability of the mouse model for use in blast research to the pelvis (Rankin et al., 2019). Whilst the current study’s findings have shown sand to be an injury mechanism at velocities encountered during blast, scaled animal models cannot be expected to be exact replicates of what occurs in humans (Bowen et al., 1968; Bowyer, 1996; Panzer et al., 2014). In this study, the resting position of the mouse prior to injury in the experimental setup is with hips abducted. This abducted starting position of the lower limbs of the mouse pre-test differs from the starting position of the dismounted soldier’s lower limb when pre-blast. The difference in these starting positions may have implications for injury thresholds, due to differences in the subsequent displacement distance of the femurs and resultant transfer of force. The mouse’s femurs rest in a near perpendicular position to the incoming blast and soil ejecta at the point of impact. This differs from the dismounted soldier’s lower limb positioning at the time of blast. It is unclear how this would affect the injury curves. One possibility is that the injury curves for traumatic amputation and pelvic fracture in the human may lie further to the right, with decreased risk of injury, due to the smaller relative surface area initially exposed to the sand blast compared to the mouse model in this study. In contrast, the lever arm and therefore moment generated about the point of injury and traumatic amputation of the femur may be relatively greater in the human compared to the mouse; this would push the injury curve to the left, with increased risk of injury. A further limitation of the mouse model that must be taken into consideration is the differences of geometry of the femoral head and acetabulum between the two species. In the mouse, the ilia are larger in the axial plane, whilst shorter in the sagittal and coronal planes. As such, the amount of bony structure in line with the loading direction superior to the acetabulum is relatively smaller than the human pelvis, which may result in a reduced amount of structural support when loading in a caudal to cranial direction. This difference may allow for a greater amount of limb flail than would be witnessed in the human and therefore increase the probability of injury. In contrast to this, the mouse femur is comparatively smaller than the human femur, accounting for only 15% of total skeletal length compared to the human femur accounting for 27% of total skeletal length (Feldesman et al., 1990; Di Masso et al., 2004). As such, in the human, a proportionally greater moment could be expected to act upon the point of initial disruption caused by high velocity sand when compared to the mouse, increasing the probability of injury. It is uncertain therefore how these data scale to the human. Irrespective of scaling, however, this study has shown that sand causes significant injury at high velocity, resulting in extensive soft tissue and skeletal disruption in the mouse model and a similar effect would therefore be expected in the human.

The experimental setup of this study, in succession with previous work utilizing a shock-tube mediated blast wave, has allowed for the injurious mechanisms of dismounted blast (primary to tertiary) to be decoupled in the mouse model (Rankin et al., 2019). Reproducing high velocity sand blast in the human is challenging due to the limitations of gas-gun systems to deliver sufficient quantities of sand; preliminary human cadaveric work may involve assessing the impact of sand blast on individual body regions or tissue types. Computational modeling could be used in combination with the results from this study to assess the effects of modified boundary conditions or mitigative strategies on injury patterns. Future research may involve investigating mitigation strategies for sand blast to the lower limbs. Military pelvic protective equipment introduced during the recent conflict in Afghanistan resulted in a reduction in the number of perineal soft tissue injuries, so similar strategies to mitigate lower limb soft tissue and skeletal injury (and, by extension, traumatic amputation) should be urgently considered (Breeze et al., 2015; Oh et al., 2015).

## Conclusion

This study is the first to replicate high velocity sand blast and the first to reproduce the pattern of injury seen in DCBI. The results suggest that sand ejecta following an explosive event can cause both soft tissue and skeletal injury alike at high velocities. Injury risk curves developed in this study showed progressively worsening severity of injuries with increasing ejecta velocity. We described a novel mechanism of injury causing traumatic amputation in the dismounted casualty which may occur independently or exacerbate those previously described. These findings implicate high velocity sand blast, in addition to limb flail, as a critical mechanism of injury in the dismounted blast casualty and these injury mechanisms should be key focuses of future research and mitigation strategies.

## Data Availability Statement

All datasets generated for this study are included in the article/Supplementary Material.

## Ethics Statement

Ethical review and approval was not required for the animal study because cadaveric mice were purchased as a by-product from Charles River UK. Male ex-breeder mice that had been already euthanized as per CRUK standard operating protocol, killed with a Schedule 1 procedure (CO_2_ asphyxiation), were subsequently used in the tests of this manuscript. These mice were accounted for under Charles River UK’s Return of Procedures Animal Use Data to the UK Home Office. As such, all animal by-product material and its use are in compliance with the UK Animal (Scientific Procedures) Act 1986.

## Author Contributions

IR, SM, and JC were involved in the conception of the study. IR, T-TN, and DC were involved in the preparation of tests, data acquisition, conducting the tests, and the data analysis. IR drafted the manuscript. All authors were involved in the interpretation of the data and revised the manuscript.

## Conflict of Interest

The authors declare that the research was conducted in the absence of any commercial or financial relationships that could be construed as a potential conflict of interest.
