# Supplementary material for: A New Understanding of the Mechanism of Injury to the Pelvis and Lower Limbs in Blast
Source: Front Bioeng Biotechnol. 2020 Aug 13;8:960. doi: 10.3389/fbioe.2020.00960 (PMC7438440; doi:10.3389/fbioe.2020.00960)
Supplement: Supplementary file 1 [file Table_1.docx]

| **Supplementary Material Table 1.** Injuries sustained by individual mice at differing sand velocities and the associated gas gun pressures | | | | | | | |
| --- | --- | --- | --- | --- | --- | --- | --- |
| Specimen | Lower limb degloving injuries | Soft tissue pelvic and perineal injuries | Traumatic amputation | Open abdominal  injuries | Pelvic fractures (tile classification) | Sand velocity  (m/s) | Gas gun pressures (reservoir / firing)  (bar) |
| 01 | - | - | - | - | - | 172 | 15 / 15.5 |
| 02 | Present | - | - | - | - | 208 | 15 / 14 |
| 03 | Present | Zone I and II | - | - | - | 223 | 18 / 17.6 |
| 04 | Present | Zone I and II | - | - | - | 207 | 15 / 14.7 |
| 05 | Present | Zone III | - | - | - | 246 | 15 / 15 |
| 06 | Present | Zone I and II | Present | - | - | 231 | 18 / 16.5 |
| 07 | Present | - | Present | - | - | 224 | 18 / 16.8 |
| 08 | Present | Zone I | - | - | - | 209 | 11 / 11 |
| 09 | - | - | - | - | - | 210 | 11 / 11.6 |
| 10 | - | - | - | - | - | 190 | 11 / 11.7 |
| 11 | Present | Zone I, II and III | Present | Present | Type C | 262 | 22 / 21.5 |
| 12 | Present | Zone I and II | Present | Present | Type C | 249 | 22 / 20.2 |
| 13 | Present | Zone I and II | Present | Present | Type C | 254 | 22 / 18.2 |
| 14 | - | Zone II | - | - | - | 174 | 7.5 / 7.2 |
| 15 | - | - | - | - | - | 166 | 7.5 / 7.3 |
| 16 | - | - | - | - | - | 179 | 7.5 / 7.2 |
| 17 | Present | Zone I, II and III | Present | Present | Type C | 252 | 22 / 18.5 |
| 18 | Present | Zone I and II | - | - | Type C | 271 | 22 / 17.5 |
| 19 | Present | Zone I, II and III | Present | Present | - | 253 | 22 / 20.1 |
| 20 | Present | Zone II | - | Present | - | 256 | 22 / 18.7 |
| 21 | Present | Zone I, II and III | - | Present | - | 260 | 22 / 21.5 |
| 22  (Control) | - | - | - | - | - | Control Specimen | 22 / 20.2 |
